# Does Single-Balloon Enteroscopy Contribute to Successful Endoscopic Retrograde Cholangiopancreatography in Patients with Surgically Altered Gastrointestinal Anatomy?

**DOI:** 10.1155/2013/214958

**Published:** 2013-05-15

**Authors:** Takuji Kawamura, Koichiro Mandai, Koji Uno, Kenjiro Yasuda

**Affiliations:** Department of Gastroenterology, Kyoto Second Red Cross Hospital, 355-5 Haruobi-cho, Kamigyo-ku, Kyoto 602-8026, Japan

## Abstract

*Background*. Balloon-assisted enteroscopy has been recognized as a useful method for performing endoscopic retrograde cholangiopancreatography in patients with complex postsurgical anatomy. *Objective*. To clarify the usefulness of single-balloon enteroscopy for performing endoscopic retrograde cholangiopancreatography successfully in patients after Billroth II gastrectomy or Roux-en-Y reconstruction and compare it with that of conventional endoscopy. *Patients and Methods*. We analyzed 204 endoscopic retrograde cholangiopancreatography procedures performed at Kyoto Second Red Cross Hospital between 1997 and 2011 in 93 patients after Billroth II gastrectomy and Roux-en-Y reconstruction with gastrectomy and choledochojejunostomy. We compared recent results with those achieved before the advent of single-balloon enteroscopy (“pre-single-balloon enteroscopy” group versus “post-single-balloon enteroscopy” group). *Results*. The rate of reaching the blind end was 11/12 (91.7%) in post-single-balloon enteroscopy Roux-en-Y gastrectomy cases and 3/9 (33.3%) in pre-single-balloon enteroscopy Roux-en-Y gastrectomy cases (*P* = 0.015). The rate of accomplishing target procedures was 7/12 (58.3%) in post-single-balloon enteroscopy Roux-en-Y gastrectomy cases. No significant difference was found in the rates for Billroth II gastrectomy cases. *Conclusion*. The single-balloon enteroscopy system is effective in reaching the blind end in patients who have undergone Roux-en-Y reconstruction; however, further innovations are needed to accomplish endoscopic retrograde cholangiopancreatography-related procedures.

## 1. Background

Endoscopic retrograde cholangiopancreatography (ERCP) is technically challenging in patients with surgically altered gastrointestinal anatomy. Double-balloon enteroscopy (DBE) [1–6] and single-balloon enteroscopy (SBE) [7–11] have been recently recognized as useful methods for performing ERCP in patients with complex postsurgical anatomy.

Before the advent of balloon-assisted enteroscopy, we mainly used conventional forward-viewing upper gastrointestinal endoscopy for performing ERCP in patients with altered gastrointestinal anatomy. The objective of this study was to compare recently achieved results of ERCP in such cases with those achieved before the advent of balloon-assisted enteroscopy.

## 2. Patients and Methods

**Figure 1:**
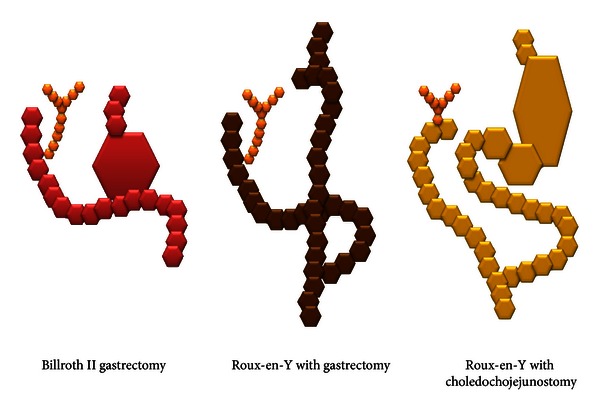
Illustrations of reconstruction surgeries.

**Figure 2:**
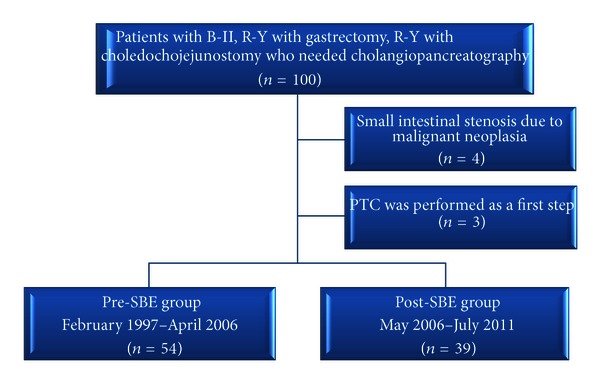
Flowchart of patients included in this study.

Between February 1997 and July 2011, we examined 100 patients who required cholangiopancreatography after Billroth II (B-II) gastrectomy, Roux-en-Y (R-Y) reconstruction with gastrectomy, and R-Y reconstruction with choledochojejunostomy (Figure 1). Four patients were excluded because of small intestinal stenosis due to malignant neoplasia, and three were excluded because percutaneous transhepatic cholangiography was performed as a first step. We divided the remaining 93 cases (204 procedures) into two groups: pre-SBE and post-SBE. The pre-SBE group consisted of patients treated from February 1997 to April 2006 (*n* = 54), and the post-SBE group consisted of patients treated from May 2006 to July 2011 (*n* = 39; Figure 2).  Table 1 shows the baseline characteristics of the patients, including the types of reconstruction surgery performed in each group.

In the pre-SBE group, we performed ERCP in all patients using a standard endoscope without a balloon overtube. In the post-SBE group, we initially used a standard endoscope for most patients and then resorted to using the SBE system in patients for whom the blind end could not be reached easily because of a long afferent loop. The SBE system consists of a video enteroscope (SIF-Q260; Olympus Medical Systems Corp, Tokyo, Japan), a sliding tube with a balloon (ST-SB1; Olympus), and a balloon controller (MAJ-1725; Olympus). The SBE system does not accept standard length accessories. Therefore, we used a part of “Conventional Component Insertion Kit” (MAJ-1420; Olympus), which has working length of 3200 mm for biliary cannulation, and disposable grasping forceps (FG-33W; Olympus), which has working length of 2500 mm for removing stones. When we must use pushing catheter to insert biliary drainage tube, we connected two conventional pushing catheters. All procedures were performed or supervised by experienced endoscopists.  Table 2 shows the endoscopes that were finally used in patients with B-II and R-Y reconstruction.

The rates of reaching the blind end, of accomplishing the target procedure, and of related complications were evaluated. “Accomplishing the target procedure” was defined as getting enhanced contrast image of the desired duct for diagnostic ERCP and providing necessary therapy for therapeutic ERCP. We used the Mann-Whitney *U* test for continuous data and a chi-square test for categorical data, except when expected cells were found to be less than 5, in which case we used Fisher's exact test.

## 3. Results

The rate of reaching the blind end in post-SBE R-Y gastrectomy cases was 11/12 (91.7%), which was higher than that in pre-SBE cases (*P* = 0.015; Table 3). On the other hand, no significant difference was observed in B-II gastrectomy cases. However, although the blind end could be reached, the target procedure could not be accomplished in some cases; therefore, the final success rate of ERCP-related procedures was 7/12 (58.3%) in post-SBE R-Y gastrectomy cases (Table 4). ERCP was never performed in the pre-SBE R-Y choledochojejunostomy group; however, the blind end was reached and the procedure was accomplished in 3/7 cases (42.9%) in the post-SBE group. The overall rate of accomplishing the target procedure was 39/54 (72.2%) in the pre-SBE group and 25/39 (64.1%) in the post-SBE group.

Table 5 lists the related complications. Perforation occurred in one pre-SBE patient during standard endoscopy and in one post-SBE patient during SBE.

## 4. Discussion

According to our single-center, retrospective analysis, SBE system was useful for reaching the blind end in patients with R-Y reconstruction; however, although the blind end could be reached, the target procedure could not be accomplished in some cases. In patients with B-II gastrectomy, high success rate was accomplished in both “pre-SBE” and “post-SBE” groups.

A side-viewing duodenoscope is usually selected for performing ERCP in patients who have not undergone surgery; however, the type of endoscope to be recommended for patients with complex postsurgical anatomy is controversial. Hintze et al. reported that they successfully reached the papilla of Vater in 92% of patients with B-II gastrojejunostomies but in only 33% of patients with R-Y anastomosis using a side-viewing duodenoscope [12]. We used a conventional forward-viewing upper gastrointestinal endoscope as the first step for ERCP in patients with complex postsurgical anatomy. As indicated in Table 3, we successfully reached the blind end in 93.3% of patients with B-II reconstruction and in 33.3% of patients with R-Y reconstruction during the “pre-SBE” period, and therefore, achieved a high success rate in patients with B-II reconstruction using a conventional endoscope without a balloon overtube. Thus, conventional upper gastrointestinal endoscopy would be the first choice for ERCP in patients with B-II reconstruction.

 DBE, which was developed by Yamamoto et al. [13], has enabled us to perform ERCP more easily in patients with altered gastrointestinal anatomy than before. SBE was developed by the Olympus Medical Systems Corp. and is considered to be a simple method because it does not have a balloon at the distal end of the enteroscope [14–16]. Recently, SBE has also been reported to be a useful method for performing ERCP in patients with complex postsurgical anatomy [7–11]. The success rate for reaching the blind end after SBE introduction in this study was clearly higher than that before SBE introduction in patients with R-Y reconstruction. May et al. reported that the complete enteroscopy rate with the SBE technique was significantly lower than that with the DBE technique [17]; however, the success rate for reaching the blind end in R-Y gastrectomy patients after the advent of the SBE technique was greater than 90% in this study. SBE could be a useful and simple method for ERCP in patients with R-Y reconstruction rather than DBE.

Nevertheless, there was no statistically significant variation in the final success rate for the accomplished target procedure between the pre- and post-SBE groups. This could be due to difficulties in cannulation of the target bile duct or pancreatic duct using SBE, because the maneuverability of the long endoscope in the deep intestine may be inferior to that of the short endoscope. In addition, even when cannulation of the target bile duct or pancreatic duct is successful, only a limited number of ERCP accessories are compatible with SBE. Shimatani et al. reported that a “short” DBE system that has a 152 cm working length can overcome this problem [3]. A “short” SBE system is not currently available; however, a balloon overtube can be modified to allow the use of a conventional forward-viewing endoscope [7]. Once the papilla is reached, the endoscope is removed from the overtube. An aperture of about 12 mm is made on the side of the overtube at a point 100 cm from its tip to allow the insertion of a conventional forward-viewing endoscope. We used this method in two cases when we performed endoscopic sphincterotomy. It is expected that a “short” SBE system or “long” ERCP accessories will be available in future.

In this study, perforation occurred as a related complication in two cases: one when a conventional endoscope was used, and the other when SBE was used. Aktas et al. reported no complications during 145 diagnostic SBE procedures [18]. Perforation occurred only after dilation of a benign stricture; therefore, SBE appears to be a safe procedure. Patients with altered gastrointestinal anatomy in whom ERCP is performed are at a high risk of developing perforation, whether SBE is used or not. Therefore, care must be taken when performing ERCP in patients with complex postsurgical anatomy.

This study has several limitations. First, this study has potential for patient selection bias by the retrospective study design. Second, the lack of defined endoscopy protocol creates heterogeneity in interventions. Third, this study has relatively small number of patients in a single center. Although the total number of patients was relatively large (*n* = 93), the patients with Roux-en-Y anatomy were only 21 cases. 

Despite these limitations, this study could indicate usefulness of SBE system in patients who have undergone Roux-en-Y reconstruction.

## 5. Conclusion

The SBE system is effective in reaching the blind end in patients who have undergone Roux-en-Y reconstruction; however, further innovations are needed to accomplish ERCP-related procedures.

## Figures and Tables

**Table 1:** Baseline characteristics of patients in the pre- and post-SBE groups.

	Pre-SBE (*n* = 54)	Post-SBE (*n* = 39)	*P* value
Age	72.0 ± 9.3	70.2 ± 13.9	0.98
Sex			
Male	37 (68.5%)	28 (71.8%)	0.73
Female	17 (31.5%)	11 (28.2%)	
Indications of ERCP			
Therapeutic ERCP	45 (83.3%)	31 (79.5%)	0.64
Biliary stones	32	17	
Malignant stenosis of the bile duct	13	13	
Benign stenosis of the bile duct	0	1	
Diagnostic ERCP	9 (16.7%)	8 (20.5%)	
Reconstruction surgeries			
Billroth II	45 (83.3%)	20 (51.3%)	<0.001
Roux-en-Y gastrectomy	9 (16.7%)	12 (30.8%)	
Roux-en-Y choledochojejunostomy	0	7 (17.9%)	

**Table 2:** Endoscopes that were chosen for Billroth-II gastrectomy or Roux-en-Y reconstruction patients.

	B-II gastrectomy		R-Y reconstruction	
	Pre-SBE (*n* = 45)	Post-SBE (*n* = 20)	Pre-SBE (*n* = 9)	Post-SBE (*n* = 19)
Conventional upper gastrointestinal endoscope (forward-viewing)	40	16	9	2
Duodenoscope (side-viewing)	2	0	0	0
Pediatric colonoscope	0	1	0	4
Push-type enteroscope	3	0	0	0
Single-balloon enteroscope	0	3	0	13

**Table 3:** The rate of reaching the blind end.

	Pre-SBE	Post-SBE	*P* value
B-II	42/45 (93.3%)	19/20 (95.0%)	0.650
R-Y gastrectomy	3/9 (33.3%)	**11/12 (91.7%)**	**0.015**
R-Y choledochojejunostomy	—	3/7 (42.9%)	—

Overall	45/54 (83.3%)	33/39 (84.6%)	0.868

**Table 4:** The rate of accomplishing the target procedure.

	Pre-SBE	Post-SBE	*P* value
B-II	36/45 (80.0%)	15/20 (75.0%)	0.746
R-Y gastrectomy	3/9 (33.3%)	7/12 (58.3%)	0.387
R-Y choledochojejunostomy	—	3/7 (42.9%)	—

Overall	39/54 (72.2%)	25/39 (64.1%)	0.404

**Table 5:** The rate of complications.

	Pre-SBE (*n* = 114)	Post-SBE (*n* = 90)
Perforation	1 (0.9%)	1 (1.1%)
Pancreatitis (moderate)	0	1 (1.1%)
Pancreatitis (mild)	3 (2.6%)	0
Post-EST bleeding	1 (0.9%)	0

## Acronyms

ERCP: Endoscopic retrograde cholangiopancreatography
DBE: double-balloon enteroscopy
SBE: single-balloon enteroscopy
B-II: Billroth II
R-Y: Roux-en-Y
PTC: percutaneous transhepatic cholangiography.
